# *Paramecium tetraurelia* chromatin assembly factor-1-like protein PtCAF-1 is involved in RNA-mediated control of DNA elimination

**DOI:** 10.1093/nar/gku874

**Published:** 2014-09-30

**Authors:** Michael Ignarski, Aditi Singh, Estienne C. Swart, Miroslav Arambasic, Pamela Y. Sandoval, Mariusz Nowacki

**Affiliations:** 1 Institute of Cell Biology, University of Bern, Baltzerstrasse 4, 3012 Bern, Switzerland; 2 Graduate School for Cellular and Biomedical Sciences, University of Bern, Freiestrasse 1, 3012 Bern, Switzerland

## Abstract

Genome-wide DNA remodelling in the ciliate *Paramecium* is ensured by RNA-mediated trans-nuclear crosstalk between the germline and the somatic genomes during sexual development. The rearrangements include elimination of transposable elements, minisatellites and tens of thousands non-coding elements called internally eliminated sequences (IESs). The trans-nuclear genome comparison process employs a distinct class of germline small RNAs (scnRNAs) that are compared against the parental somatic genome to select the germline-specific subset of scnRNAs that subsequently target DNA elimination in the progeny genome. Only a handful of proteins involved in this process have been identified so far and the mechanism of DNA targeting is unknown. Here we describe chromatin assembly factor-1-like protein (PtCAF-1), which we show is required for the survival of sexual progeny and localizes first in the parental and later in the newly developing macronucleus. Gene silencing shows that PtCAF-1 is required for the elimination of transposable elements and a subset of IESs. *PTCAF-1* depletion also impairs the selection of germline-specific scnRNAs during development. We identify specific histone modifications appearing during *Paramecium* development which are strongly reduced in *PTCAF-1* depleted cells. Our results demonstrate the importance of PtCAF-1 for the epigenetic trans-nuclear cross-talk mechanism.

## INTRODUCTION

The epigenetic influence of maternal cells on the development of their progeny has long been studied in eukaryotes (1–4). Multicellular organisms provide their zygotes not only with nutrients but also with functional elements required for proper development, such as coding and non-coding RNAs. These maternally deposited RNAs exhibit a variety of functions, from regulating gene expression to assuring genome integrity (5–10). However, the epigenetic influence of maternal RNAs on the development is not restricted to multicellular eukaryotes.

In single-celled ciliates these RNAs participate in programming of large-scale DNA deletion and genome rearrangements during development. One of the characteristic features of ciliates is the decoupling of germline and somatic functions into two distinct types of nuclei, the diploid micronucleus (MIC) and the polyploid macronucleus (MAC). Meiosis of the germline MICs initiates sexual events and after fertilization, new MICs and MACs develop from the zygotic nucleus. Concurrently, the old MAC is degraded and replaced by the new one. During this process the old MAC undergoes morphological changes, losing its usual shape and forming a skein, which eventually breaks down into fragments. These fragments are diluted out and disappear from the cytoplasm after several vegetative divisions following sexual reproduction. Before entering the vegetative life cycle, the cell goes through a karyonidal division that distributes the two newly formed MACs between two daughter cells (11). The diploid MIC is transcriptionally silent during vegetative growth and propagates the germline over sexual generations. During sexual reproduction the two MICs present in *Paramecium tetraurelia* undergo meiosis, generating eight haploid products, seven of which immediately degrade. The surviving gamete then divides mitotically. In the case of self-fertilization (autogamy) the two mitotic products fuse to form a zygotic nucleus. The zygotic nucleus undergoes two subsequent mitotic divisions generating four diploid nuclei. Two of these nuclei remain diploid and unaltered and become the new MICs. The other two nuclei develop into the new MACs through a massive DNA polyploidization, chromosome fragmentation and programmed DNA elimination (12). During this process the germline and the somatic genomes are compared using scnRNAs originated from the MIC, and long non-coding RNAs (ncRNAs) from the parental MAC. Current experimental data proposes that scnRNAs may be compared to an RNA copy of the parental MAC genome in the form of nascent transcripts, rather than to the MAC DNA itself (13,14). This comparison is necessary to identify germline-specific sequences such as transposable elements, minisatellites, as well as ∼45 000 internal eliminated sequences (IESs) (15). These sequences are then eliminated by imprecise (transposable elements and minisatellites) or precise (IESs) excision mechanisms reducing the MAC genome down to coding and regulatory sequences. Imprecise removal of repetitive and genome invading sequences leads to the fragmentation of chromosomes into smaller, *de* *novo* telomerized molecules (16). Since IESs are present in coding as well as non-coding sequences precise excision is necessary to maintain their integrity. *P. tetraurelia* IESs are between 26 and 5316 bp long, are typically unique sequences, non-coding elements that are always flanked by two 5′-TA-3′ dinucleotides, of which one remains in the MAC genome after IES excision (17). Approximately one-third of tested IESs present in *P. tetraurelia* have been shown to be under homology-dependent maternal control (18). It has been previously demonstrated that the introduction of an IES sequence into the vegetative parental MAC by microinjection prevents the excision of the complementary IES from the developing MAC genome (19). IESs affected by this kind of manipulation are termed maternally controlled IESs (mcIES). The remaining two-thirds of the IES are not affected and are therefore called non-maternally controlled IESs (non-mcIES).

Recent advances have shown a requirement for meiosis-specific Dicer- and Piwi-like proteins in the accumulation of scnRNAs (scan RNAs), a class of 25-nt small RNAs (sRNAs) produced from meiotic micronuclei (20–24). High-throughput mapping of scnRNAs in *Tetrahymena* demonstrate they are enriched at IES sequences (25). In *Paramecium*, scnRNAs are produced by a pair of Dicer-like protein paralogs (Dcl2 and Dcl3) (21) and become associated with a pair of Piwi-like protein paralogs (Ptiwi01 and Ptiwi09) (20). As MIC-specific sequences are not present in the parental MAC, scnRNAs complementary to these sequences cannot pair in the parental MAC and are further transported to the developing MAC. Here they target MIC-specific sequences for excision by the domesticated transposase PiggyMac (26). The transport of scnRNAs from the parental to the developing MAC is believed to be accompanied by Nowa1/Nowa2 proteins (27). In addition, Nowa1 and Nowa2, which contain both putative RNA- and Argonaute-binding domains (27), are thought to mediate interactions between long ncRNAs and scnRNAs carried by Ptiwi01/09. Recently a new class of development-specific, IES-matching small RNAs (iesRNAs) has been identified (28). iesRNAs, which are ∼21–31 nt long (mode of 27 nt), were proposed to be produced from excised IESs by Dicer-like protein 5 (Dcl5) and may amplify the sRNA signal needed for complete IES removal.

**Figure 1.**
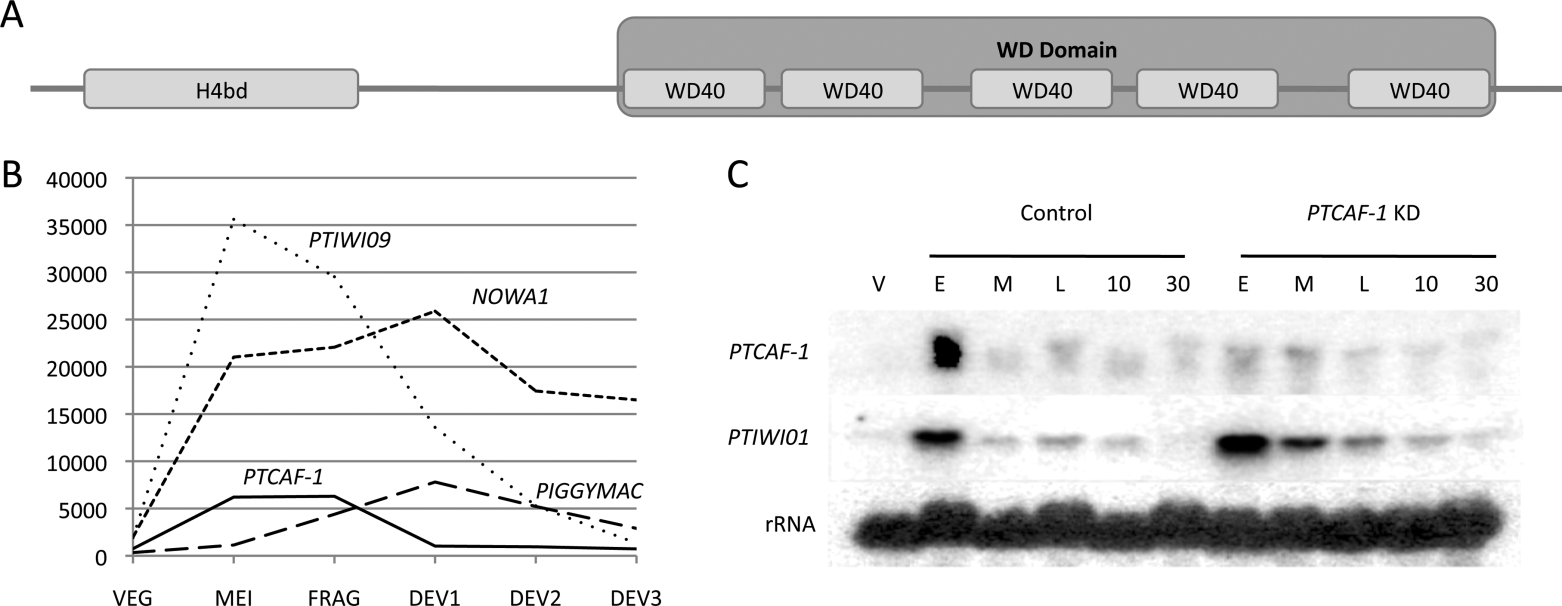
PtCAF-1 structure and expression pattern. (**A**) Schematic drawing of the PtCAF-1 domain architecture. The 391 amino acid long protein has both a histone binding domain (H4bd) between amino acids 15 and 84 and five WD40 repeats between the amino acids 152 and 370. The five WD40 repeats fold to form a WD domain. (**B**) Expression of *PTCAF-1* based on microarray data from (29,36) during sexual reproduction of *Paramecium tetraurelia* in comparison to *NOWA1*, *PTIWI09* and *PIGGYMAC*. VEG: vegetative cells; MEI: beginning of macronuclear fragmentation and micronuclear meiosis; FRG: population in which about 50% of cells have a fragmented old macronucleus; DEV1: earliest stage at which a significant proportion of cells has visible macronuclear anlagen; DEV2: majority of cells with macronuclear anlagen; DEV3: population of cells 10 h after DEV2. (**C**) Expression of *PTCAF-1* and *PTIWI01* analysed by northern blot. Cells during vegetative growth (V) and during autogamy where sampled from control and *PTCAF-1* KD cultures and analysed for *PTCAF-1* and *Ptiwi01* expression. E indicates a culture with 50% of cells with fragmented MAC (E ≈ FRG in B). M indicates a culture with 100% of cells with fragmented MAC (M ≈ between FRG and DEV1 in B). L indicates a culture 6 h after M (L ≈ DEV1 in (B)). 10 and 30 indicate cultures 10 and 30 h after M, respectively (10 ≈ DEV2 in B, 30 is ∼10 h later than DEV3 in B). Consecutive hybridizations on one membrane were performed with *PTCAF-1*, *PTIWI01* and 17S rRNA probes.

We selected *P. tetraurelia* chromatin assembly factor 1 subunit C-like protein (PtCAF-1) based on a microarray dataset (29), in which it showed substantial upregulation during autogamy. PtCAF-1 is a 391 amino acid long protein, with a N-terminal histone binding domain (H4bd) while its C-terminal is comprised of five WD40 repeats, which, in other organisms, are known to fold together to form the WD40 domain (30) (Figure 1A). Proteins containing these types of domains belong to the CAF1C_H4-bd super-family (31) known to associate with chromatin assembly and remodelling factors by the direct binding to histone H3 and H4 (32). In humans the chromatin assembly factor (CAF-1) complex is required for chromatin assembly following DNA replication and DNA repair (33). A groove formed between the alpha helical H4bd and an extended loop protruding from the beta-propeller structure of the WD40 domain binds histone H4 in the human subunit C of CAF-1 complex (34). The WD40 domain forms a rigid scaffold, comprised of beta sheet repeats, for coordinated multi-protein interactions (30). Specificity for interactions is brought about by the amino acids present in loops on the outside of the repeats (35).

In *Tetrahymena thermophila*, it has been shown that histone H3 lysine 9 trimethylation and H3 lysine 27 trimethylation (H3K9me3/H3K27me3) are associated with programmed DNA elimination. It has been demonstrated that Twi1p (23), a Piwi homologue (37), in association with scnRNAs recruits Ezl1p (38), an E(z) homologue (39) to MIC-specific sequences in the developing MACs. Ezl1p trimethylates H3K27 at these loci which in turn leads to trimethylation of H3K9. Chromodomain containing proteins, Pdd1p/Pdd3p are recruited by these modifications and mediate chromatin condensation (40,41). This MIC-specific heterochromatin precedes DNA elimination (38,42).

Here we identify H3K9me3 and H3K27me3 to be histone modifications associated with DNA elimination in *Paramecium*. Furthermore, we show that H3K9me3 and H3K27me3 are dependent on the presence of PtCAF-1-like protein as well as the previously characterized Nowa1 protein. Our analyses of IES retention following depletion of PtCAF-1 suggest that PtCAF-1 is a new protein involved in epigenetic programming of genome rearrangements in *P. tetraurelia*.

## MATERIALS AND METHODS

### *Paramecium* strains and cultivation

Experiments were performed on the homozygous mating-type 7 of stain 51. Cells were grown in wheat grass powder medium (WGP; Pines International, Lawrence, KS) bacterized with *Klebsiella pneumoniae* and supplemented with 0.8mg/l of β-sitosterol (Merck). Cultivation was carried out at 27°C as previously described (43).

### Constructs

PtCAF-1 (Genbank accession: XM_001457471) sequence with upstream and downstream regulatory elements extending up to the next coding sequences was cloned into pGEM-T Vector (Promega). To obtain the N-terminally tagged version of PtCAF-1, Enhanced Green Fluorescent Protein (EGFP) optimized for *Paramecium* codon usage was inserted after the initiating ATG of PtCAF-1. *PO1* (Genbank accession: XM_001427355). *PO2* (Genbank accession: XM_001461420).

### Gene silencing

Silencing during autogamy was performed as previously described (44). To deplet cells of PtCAF-1 a 735-bp fragment of the protein coding sequence was cloned between two inverted T7 promoters of the L4440 vector. This vector was transformed into the HT115 (DE3) *Escherichia coli* strain. This *E.coli* strain was used to induce RNAi by feeding. For the control cultures an empty L4440 vector expressing dsRNA corresponding to the multiple cloning site was used. For *NOWA1*, *PTIWI01/09* and *DCL2/3* knockdowns previously described silencing construct were used (20–21,27).

### Survival test

After finishing development in silencing medium, 30 cells were placed in individual depressions with regular bacterized medium and monitored for survival during three consecutive days.

### DNA and RNA extraction

Genomic DNA was isolated from 10 ml cultures at various time points during development according to GenElute Mammalian Genomic DNA Miniprep Kit (Sigma-Aldrich). Whole-cell RNA extracts were prepared from 400 ml of culture following the TRI Reagent BD protocol (Sigma-Aldrich) and resuspended in RNase free water.

### Southern and northern blots

DNA and RNA electrophoresis were carried out following standard procedures, respectively (45,46). After depurination in 0.25-M HCL, DNA (5 μg per sample) was transferred in 0.4-M NaOH from agarose gels to Hybond N+ membranes (GE Healthcare Life Sciences). RNA (10 μg per sample) was transferred from denaturating-agarose gels containing 2.2-M formaldehyde to Hybond N+ membrane in 20x saline-sodium citrate (SSC) buffer and crosslinked by UV illumination (UV Stratalinker, Stratagene). Probes were labelled with α-^32^P dATP (3000 Ci/mmol) using RadPrime DNA Labelling System (Invitrogen). Hybridization was performed in Church Buffer (1% bovine serum albumin (BSA), 1-mM ethylenediaminetetraacetic acid, 0.5-M NaPO4 pH 7.2, 7% SDS) at 60°C over night. Prior to exposure membranes were washed for 30 min in 2x SSC, 0.1% SDS at 60°C. Exposed screens were scanned with Storm 860 (Amersham), the intensities where quantified using ImageJ (http://rsb.info.nih.gov/ij/).

### RNA sequencing

Enrichment of small RNAs was achieved by using the mirVana miRNA isolation Kit (Ambion). Small RNA libraries were generated and sequenced in accordance to Illumina protocols (TruSeq Small RNA, Illumina). Raw sRNA sequences have been deposited in the European Nucleotide Archive under the accession number PRJEB4978 for PtCAF-1. Reads for early time point run can be retrieved from the accessions: ERR372239–ERR372250 and for late time point run from the accessions: ERR372001–ERR372007. For the control, raw sRNA sequences were previously deposited under the accession number SRR907874 for the early time point and under SRR907875 for the late time point.

### PCR analysis of IES retention

According to suppliers manual GoTaq polymerase (Promega) was used for IES retention polymerase chain reactions (PCRs) according to supplier's manual. The primer sequences are available in the Supplemental Material (Supplementary Table S1).

### Macronuclear transformation

Transformation of the macronucleus was achieved by microinjection (47). PtCAF-1 with 5′ and 3′ adjoining regulatory sequences was cloned into pGEMT vector and tagged N-terminally with Green Fluorescent Protein (GFP). The linearized plasmid was injected into the vegetative MAC of *P.tetraurelia*. Transformed clones were determined by dot-blot analysis or detection of GFP expression.

### GFP localization

Cells injected with GFP tagged PtCAF-1 were grown to high density (3000 cells/ml). Small samples were removed at various life cycle stages and counterstained with 4′,6-diamidino-2-phenylindole (DAPI). GFP localization was observed by fluorescence microscopy (Leica AF6000 system).

### Immunofluorescence

Cell fixation and staining conditions were adapted from (48). Cells were permeabilized with Pipes–Hepes–EGTA–MgCl_2_ (PHEM) buffer with 1% Triton X-100 and subsequently fixed in PHEM with 2% paraformaldehyde. Blocking was carried out in Tris-buffered saline with 10 mM EGTA and 2 mM MgCl_2_ (TBSTEM) with 3% BSA. Cells were stained with rabbit anti-H3K9me3 (1:200, Millipore, cat: 07-442, lot: 2120113) and anti-H3K27me3 (1:200, Millipore, cat: 07-449, lot: 2275589) primary-antibodies. Alexa Fluor 488 conjugated goat anti-rabbit secondary antibody (1:200, Life Technologies, cat: A11008, lot: 1275894) was used for detection. Stained cells were mounted onto glass slides and imaged on the Leica AF6000 system with a HCX PL Fluortar 100.0 × 1.30 oil objective.

### Confocal Microscopy

*Paramecium* cells were permeabilized for 20 min in PHEM buffer with 1% Triton X-100, fixed in 2% paraformaldehyde for 10 min and denatured with 0.1-M HCl for 5 min. The cells were then washed with phosphate buffered saline (PBS) for 5 min and blocked for 1 h in TBSTEM buffer with 3% BSA. After blocking the cells were incubated with the primary antibody (rabbit anti-H3K27me3, 1:100, Millipore, cat: 07-449, lot: 2275589 or rabbit anti-H3K4me3, 1:500, Abcam, cat: ab8580, lot: GR144288-1) diluted in TBSTEM buffer with 3% BSA + 0.1% Triton X-100 for 1 h and then washed three times for 10 min with PBS. This was followed by secondary antibody incubation (1:500 goat anti-rabbit conjugated with AlexaFluor 568 or 1:200 goat anti-rabbit conjugated with AlexaFluor 488) at 37°C for 1.5 h. The cells were again washed three times for 10 min with PBS and then stained with DAPI (1 mg/ml) for 5 min. The cells were then washed once with PBS and mounted with Prolong^®^ Gold Antifade mounting medium (Life Technologies) on a glass slip sealed with a coverslip. Imaging was performed with Olympus FLUOVIEW FV1000 confocal laser scanning microscope and images were processed with Imaris software (Bitplane).

## RESULTS

### *PTCAF-1* expression coincides with the onset of sexual reproduction

During meiosis and subsequent fragmentation of the parental MAC the *PTCAF-1* gene is up regulated, coinciding with the expression of known genes involved in genome rearrangements (Figure 1B). In addition to *PTCAF-1* there are two ohnologs (paralogous sequences derived from *Paramecium* whole-genome duplications (WGD)), *PTCAF-1* ohnolog 1 (*PO1*) and *PTCAF-1* ohnolog 2 (*PO2*). PO1 and PO2 proteins are 94% identical and both share 68% identity with PtCAF1. According to microarray data (36) both *PO1* and *PO2* are expressed at low levels throughout the entire life cycle of *P. tetraurelia* (Supplementary Figure S1A) and are not upregulated during sexual development and *PTCAF-1* silencing does not affect expression of *PO1* and *PO2* (Supplementary Figure S1B). To confirm the expression of *PTCAF-1* we examined total RNA samples taken at specific life cycle time points for the presence of *PTCAF-1* mRNA by northern-blot analysis (Figure 1C). Consistent with the microarray data, the analysis of the RNA sample taken during vegetative growth (V) showed only a very weak expression of *PTCAF-1*. In the samples taken at consecutive time points during autogamy from the control culture, an elevated *PTCAF-1* signal was detected in the early (E) stage of autogamy after which the expression dropped to above vegetative levels at the middle time point (M) and was maintained for the next 30 h until the completion of new MAC development.

### Knockdown of *PTCAF-1* impairs cell survival and DNA elimination

**Figure 2.**
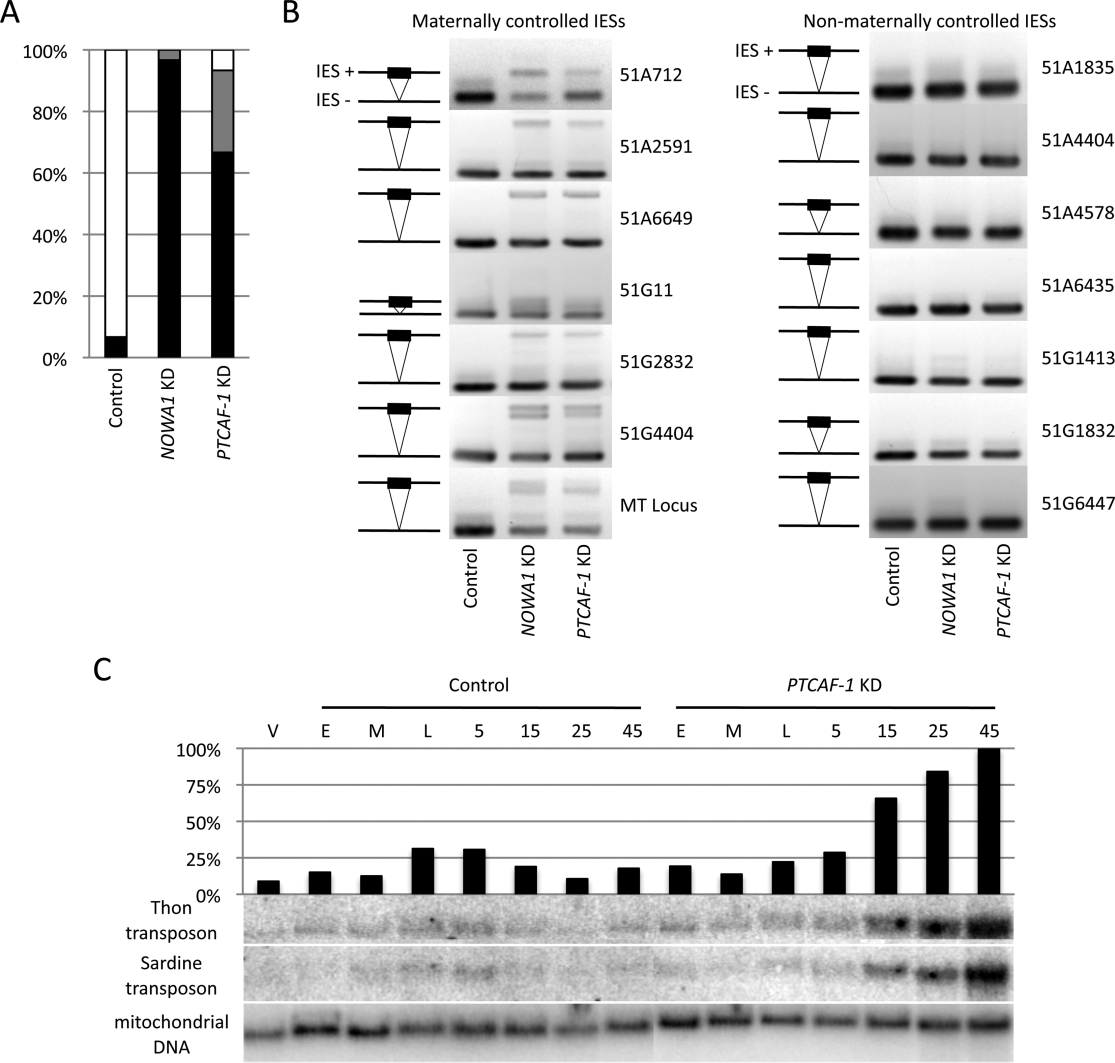
Effect of *PTCAF-1* knockdown on cell survival, IES and transposon retention. (**A**) Effect of *PTCAF-1* knockdown on cell survival. After going through autogamy in silencing medium the cells where recovered in WGP medium. The graph shows the survival after three days. Black: percentage of dead cells; grey: percentage of sick cells showing an altered number of divisions or behavior; white: percentage of cell growing at a normal rate. *NOWA1* KD was used as a positive control. Silencing vector without insert was used as negative control. (**B**) Effect of *PTCAF-1* knockdown on IES excision. IES retention was tested by PCR using primers flanking specific IESs sequences within the *51G* and *51A* genes as well as the mating-type locus (MT Locus). The excised form (IES−) is always detectable due to its presence in the fragments of the parental MAC. The unexcised form (IES+) is only detectable in case of retention in the newly developing macronuclei. Maternally controlled IESs: 51A712, 51A2591, 51A6649, 51G11, 51G2832, 51G4404, MT-Locus; non-maternally controlled IESs: 51A1835, 51A4404, 51A4578, 51A6435, 51G1413, 51G1832, 51G6447. (**C**) Effect of *PTCAF-1* KD on Thon and Sardine transposons. Total DNA was extracted from vegetative (V), control and *PTCAF-1* KD cultures at specific time points during sexual reproduction. E, M, L as in Figure 1C; 5, 15, 25 and 45 h after L. After running the DNA on a 1% agarose-gel and transfer to nylon membrane the samples were hybridized with Sardine and Thon transposon-specific probes. A mitochondrial DNA-specific probe was used as loading control. The graph shows the Thon signal quantification normalized to the loading control.

Upon *PTCAF-1* knockdown (*PTCAF-1* KD) we observed a strong decrease of *PTCAF-1* mRNA level in the early stages of autogamy (E) compared to control (Figure 1C). To assess whether PtCAF-1 might play a role in *Paramecium* sexual development, we tested the survival of sexual progeny after *PTCAF-1* KD (Figure 2A). Sixty-seven percent of the *PTCAF-1* KD cells in our survival test died during a time course of 3 days (up to 12 cell divisions) after the progeny cells were refed and allowed to divide vegetatively (Figure 2A). Twenty-seven percent of the cells did not divide at the normal rate of four divisions per 24 h and 6% showed no growth defects. In the control culture, 94% of the cells grew at normal rates while 6% died. In the positive control (*NOWA1* KD), 97% of the cells died and 3% survived with a reduced growth rate. This result was confirmed by using RNAi construct targeting an alternative region of *PTCAF-1* (Supplementary Figure S2A). No cell death or reduced division was observed for *Paramecium* cells grown vegetatively in silencing medium for more than 12 divisions. *PTCAF-1* KD did not result in premature termination of development at an early stage as we were able to observe all the developmental stages and the final formation of new developing MACs (data not shown). We saw no structural irregularities during development and there was no notable differences in DNA content between the *PTCAF-1* KD cells and the control cells. Our deep sequencing results suggest that the overall scnRNA levels that are detected at the early time point during development are not affected in *PTCAF-1* KD compared to control (Figure 4B). This suggests that the expression and functions of Dcl2/3 and Ptiwi01/09 (responsible for the production and protection of scnRNAs) were not affected by *PTCAF-1* KD. We confirmed by northern blot that the expression of *PTIWI01* is not affected by *PTCAF-1* KD (Figure 1C), and that the localization of Ptiwi09-GFP is unchanged by this knockdown (data not shown).

**Figure 3.**
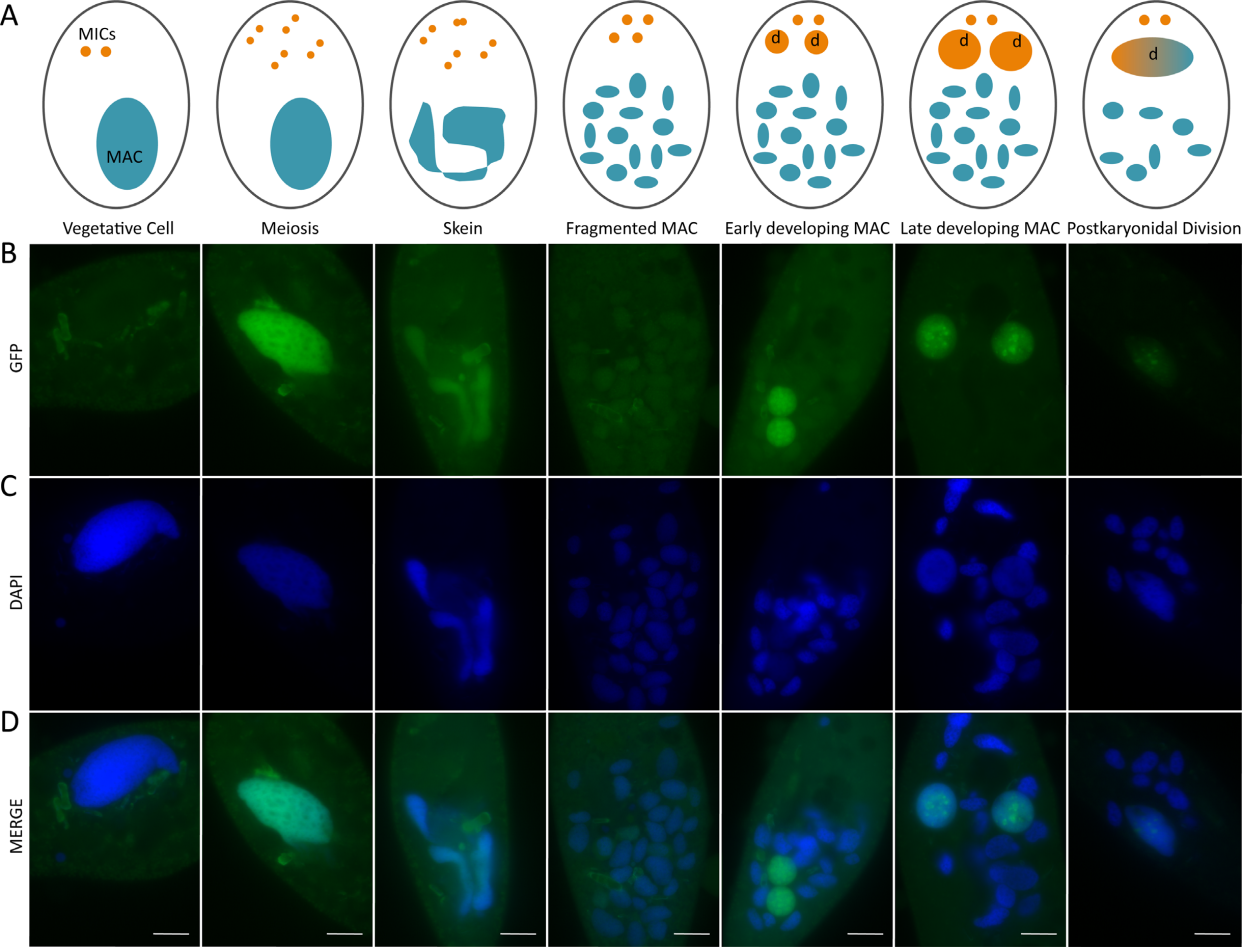
Localisation of PtCAF-1 during sexual reproduction by GFP-fusion. PtCAF-1 tagged N-terminally with GFP was expressed at the onset of meiosis and localized in the parental MAC where it remained during skein formation and fragmentation. It later localized in the developing MACs and was present in the new MAC after karyonidal division. (**A**) Schematic drawing of the life cycle stages of *Paramecium tetraurelia*. Blue: parental macronucleus; orange: micronuclei, marked with (d) when determined to develop into new MAC. (**B**) GFP signal. (**C**) DAPI signal. (**D**) Merged signals; scale bar: 10 μm.

**Figure 4.**
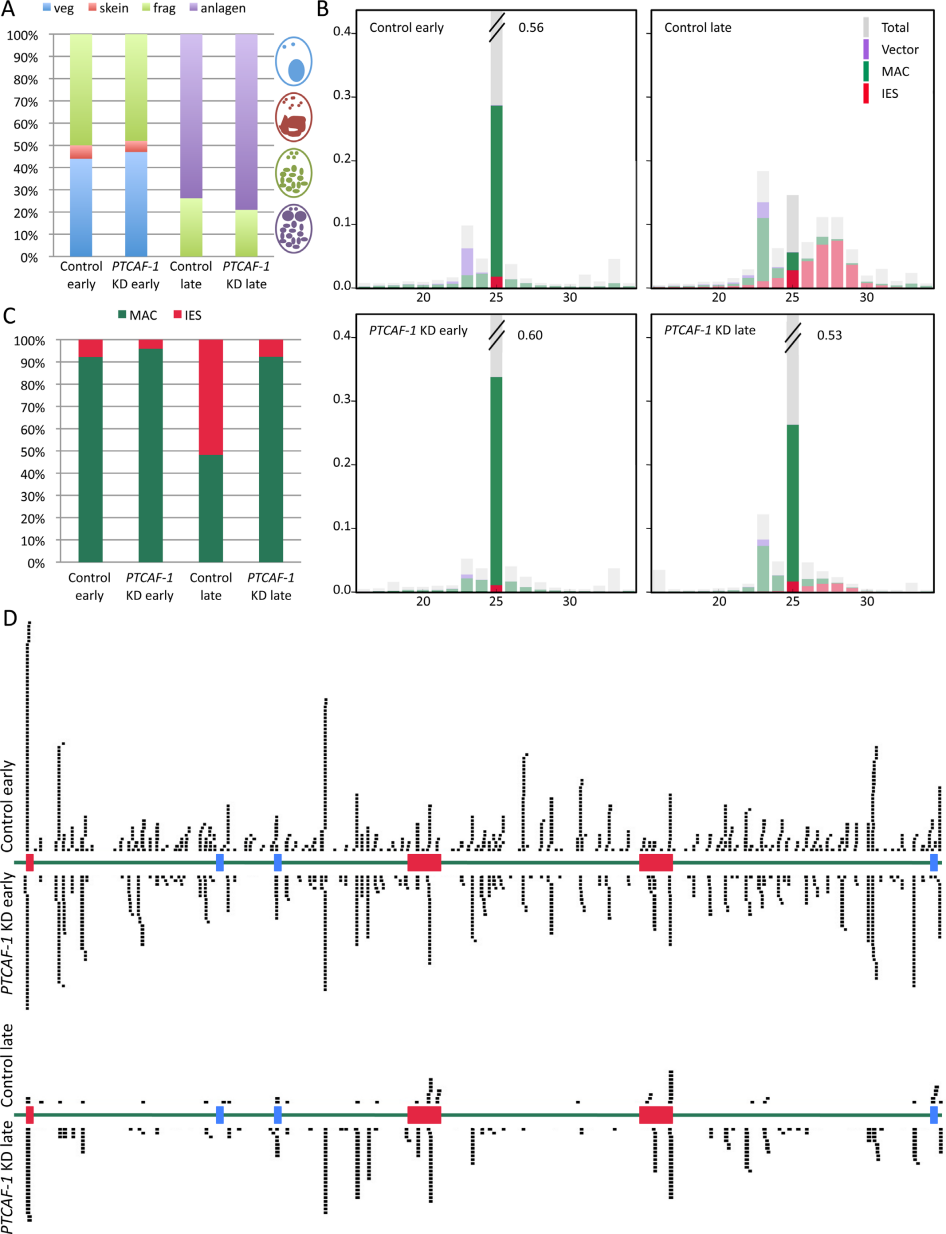
Effect of *PTCAF-1* silencing on scnRNAs. (**A**) Distribution of life cycle stages in the *Paramecium* culture samples used for smallRNA libraries. RNA of an early and a late time point of a *PTCAF-1* KD and a control culture was extracted and smallRNA libraries were prepared. Early time point samples contain cells in the vegetative (blue), skein (red) and fragmented (green) stage. Late time points contain cells in the fragmented (green) or late fragmented stage with visible developing MACs (purple). (**B**) Distribution of total 16 to 34 nucleotide small RNA library reads (Total/grey), mapped to the macronuclear genome (MAC/green), internal eliminated sequences (IES/red) and the plasmid backbone used for silencing (Vector/purple) (the same pair of histograms was previously used as the control in (28). (**C**) Effect of *PTCAF-1* silencing on 25-nt scnRNA. Extraction of 25-nt reads mapping to MAC (green) and to IESs (red). (**D**) 25-nt scnRNAs reads mapped to the *51G* locus. An extract of the *51G* gene locus (green) containing three mcIES (red) and three non-mcIES (blue) was mapped with 25-nt scnRNA reads (black) from the early and late time point of *PTCAF-1* silenced and control culture. The reads are mirrored over the corresponding sequence.

To assess whether *PTCAF-1* KD affects DNA elimination, we tested for IES retention in the newly developed MACs by PCR (Figure 2B). Primers flanking 14 known IESs were used (Supplementary Table S1). In the case of correct IES excisiona shorter fragment (IES−) was amplified. If an IES was retained in the genome the amplicon was longer (IES+). In this assay, the *PTCAF-1* KD impairs the elimination of mcIES from the developing MAC but does not affect the excision of non-mcIES. This result was confirmed by using RNAi construct targeting an alternative region of *PTCAF-1* (Supplementary Figure S2B). To verify if PtCAF-1 is also involved in the elimination of other DNA elements from the developing MAC genome we assessed whether the Thon and Sardine transposons were retained in *PTCAF-1* KD cells by Southern blot analysis (Figure 2C). Samples of genomic DNA were extracted from consecutive time points during autogamy from a *PTCAF-1* KD and a control culture. In the control culture a temporary accumulation of both transposable elements in the intermediate time points (L,5) was detected (Figure 2C). This result is concordant with previous results in wild-type cells, where the accumulation of MIC-specific sequences is detected transiently at intermediate time points, followed by their removal later during development when DNA elimination starts in the developing MACs (49). In *PTCAF-1* KD the accumulation of Thon and Sardine sequences start at the same intermediate stages but in contrast to the control continues until late in development (Figure 2C). The amount of accumulating signal is eventually more than four times that observed in the control suggesting that sequences are not removed from the MAC genome and are amplified with the chromosomes to high ploidy.

### PtCAF-1 localizes in the parental and the developing MAC

To determine the localization of the PtCAF-1 protein during development, *P. tetraurelia* was transformed with an N-terminal GFP tagged *PTCAF-1* set under the control of a sequence region containing the putative endogenous *PTCAF-1* promoter. The localization of PtCAF-1 GFP fusion protein was observed at distinct stages during autogamy by fluorescence microscopy (Figure 3). No GFP signal was detectable during vegetative growth. Upon meiosis the GFP fusion protein was expressed and localized in the parental MAC where it remained during skein formation and in the separated fragments. Later in development the fusion protein accumulated in the new MACs where it persisted throughout MAC development. It was still detectable in the new MACs after karyonidal division. The PtCAF-1 GFP signal decreased with the maturation of the developing MACs. This localization pattern strongly resembles that of Nowa1 (27) reinforcing the hypothesis of PtCAF-1 being a factor in genome rearrangements of *P. tetraurelia*.

### *PTCAF-1* knockdown affects RNA scanning by preventing the elimination of MAC-specific scnRNAs

High-throughput sequencing of *P.tetraurelia* small RNAs was performed to determine whether the silencing of *PTCAF-1* might affect scnRNA levels during development. Small RNAs extracted from two developmental time points in control and *PTCAF-1* KD cultures were sequenced. In both early time points about 50% of the cells contained fragmented parental MACs while the rest showed intact parental MACs or the beginning of MAC fragmentation. In the late time point all cells in both the cultures had fragmented parental MACs and about 80% showed enlarged developing new MACs (Figure 4A). As previously shown (Figure 4B is a slightly modified version of the histograms in Figure 3A of (28)), the amount of 25-nt sRNAs corresponding to MAC genome-specific sequences (green) strongly decreased in the control from early to late time point which we suggested is due to the progression of scanning (28). In the *PTCAF-1* KD this effect was suppressed suggesting that in the absence of PtCAF-1 the RNA scanning process was perturbed. When comparing only the 25 nt reads matching MAC genome- and IES-specific sequences (Figure 4C) it becomes apparent to what degree *PTCAF-1* KD disturbed the elimination of scnRNAs mapping to MAC genome-specific sequences. In the control MAC genome-specific reads accounted to over 90% of the reads in the early time point while in the late time point their proportion dropped to under 50%. In contrast, in *PTCAF-1* KD the amount of MAC-mapping reads remained above 90% in the late time point. This suggests that PtCAF-1 is necessary for the elimination of MAC genome-matching scnRNAs from the scnRNA pool during development. A second observable effect of *PTCAF-1* KD is the decrease of iesRNAs (∼26–30 nt) in the late time point (Figure 4B). While in wild-type cells iesRNAs are produced from already excised IESs, *PTCAF-1* KD blocks the elimination of mcIESs therefore the production of iesRNA is strongly reduced. Compared to the late time point of the control, in the late time point of *PTCAF-1* KD, only a small number of IES matching reads (red) have been detected.

To graphically illustrate the effect of *PTCAF-1* KD on scnRNAs at a specific genomic locus, a region of the micronuclear sequence of the *51G* surface antigen was selected (Figure 4D). The region consists mostly of MAC genome-specific sequences (green) and contains six IESs, three mcIESs (red) and three non-mcIESs (blue). In the early time point of control and *PTCAF-1* KD sample 25 nt reads mapped over the entire *51G* region with no visible preference for MAC- or IES-specific sequences. This suggests that up to this point in development there is no selection between MAC- and IES-specific scnRNAs. To be able to compare amounts of scnRNAs of individual libraries we normalized the mapped reads to reads per million total reads (r/m) (Supplementary Table S2). We noticed that in the early time point of the control sample 22.1 r/m mapped within the *51G* region while in the late time point only 2.8 r/m mapped to the same region. In addition to the obvious reduction of the amount of 25 nt reads, more than half of the reads in the late time point mapped concentrated at IES-specific sequences. This strong reduction of the total amount of 25 nt reads and the selection of IES-specific scnRNAs is in agreement with the current scnRNA model where MAC-specific scnRNA are removed from the pool and only the IES-specific scnRNAs persist into the late stages of development. In the early time point of the *PTCAF-1* KD culture 24.6 r/m mapped to the *51G* region, a similar amount to the early time point of the control. Interestingly, in the late time point of *PTCAF-1* KD there were still 18.3 r/m mapping to the *51G* region and these reads were not exclusively concentrated at IES sequences. In summary, our sRNA analyses show that as a consequence of the *PTCAF-1* KD the removal of MAC genome-specific scnRNAs during development is impaired.

To exclude the possibility that the observed aberrations during scanning in *PTCAF-1* KD cells are due to changes in the transcription of scnRNA presursors in the meiotic MICs or long non-coding RNAs in the parental MAC, we performed RT-qPCR on IES- and MAC-specific non-coding transcripts at an early time point during development. We designed probes for an intergenic locus (non-IES) and for an IES sequence (IES 51A4578). The results were normalized to two housekeeping genes. There is no significant difference in the amount of transcripts produced from either of the loci between control and *PTCAF-1* KD (Supplementary Figure S3). These results show that the scanning defect in *PTCAF-1* KD is not attributed to the lack of non-coding transcripts produced from the parental MAC nor due to an effect on the production of scnRNAs precursors (which is consistent with our sRNA sequencing result).

### *PTCAF-1* knockdown reduces the levels of H3K9me3 and H3K27me3 during development

**Figure 5.**
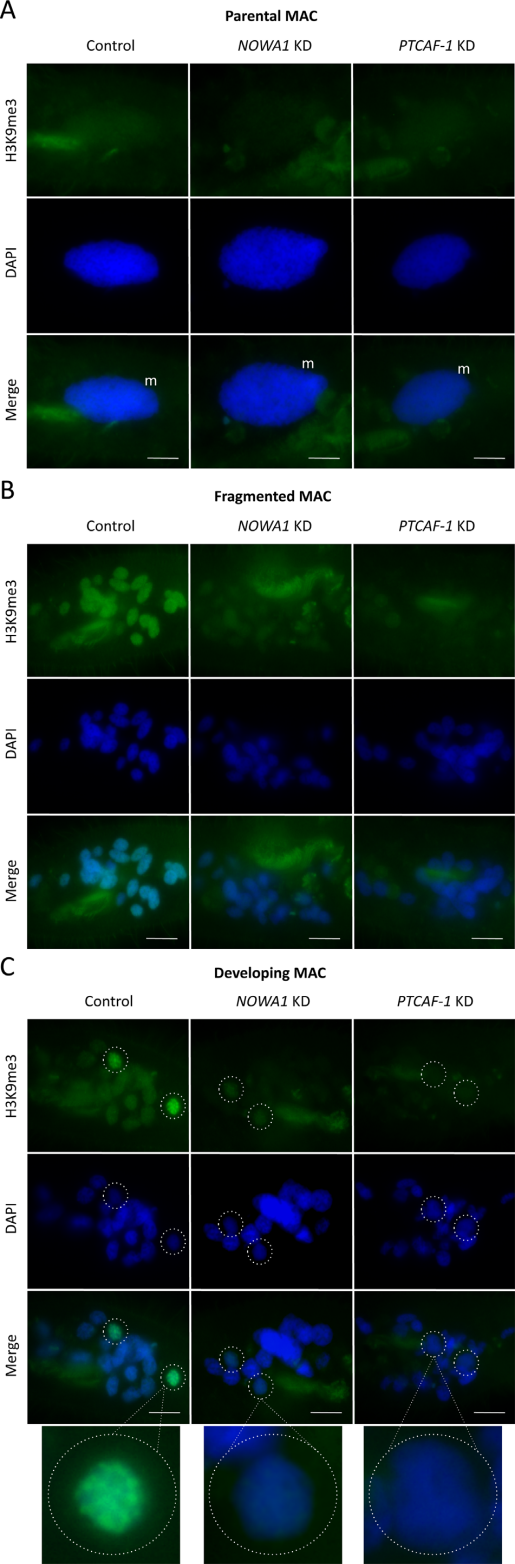
Comparison of H3K9me3 signals between control, *PTCAF-1* KD and *NOWA1* KD cells. (**A**) H3K9me3 signal in the parental MAC. (**B**) H3K9me3 signal in the parental MAC after fragmentation. (**C**) H3K9me3 signal in the developing MACs. Green: H3K9me3; blue: DAPI; m: parental macronucleus; dotted circles: developing macronucleus; scale bar: 10 μm. Control: cells silenced with empty L4440 vector, *NOWA1* KD: *NOWA1* knockdown, *PTCAF-1* KD: *PTCAF-1* knockdown.

**Figure 6.**
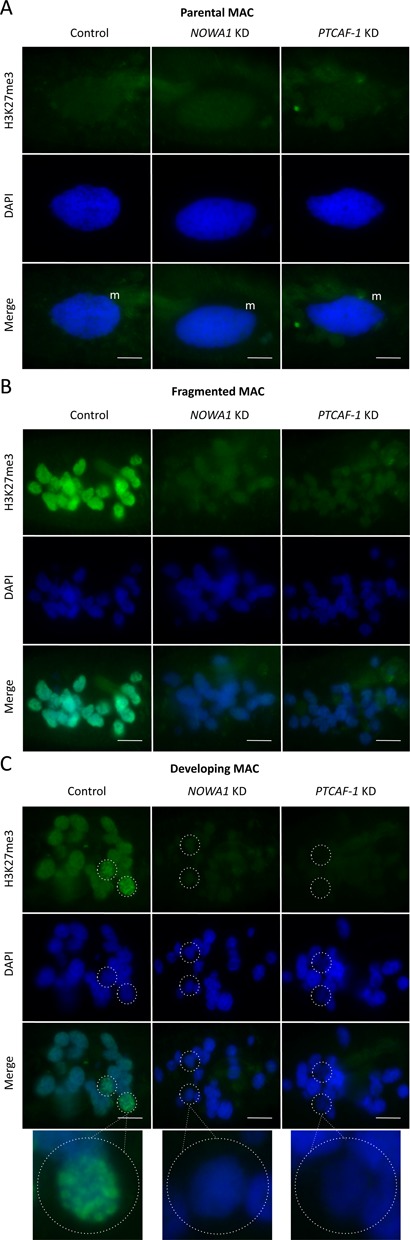
Comparison of H3K27me3 signals between control, *PTCAF-1* KD and *NOWA1* KD cells. (**A**) H3K27me3 signal in the parental MAC. (**B**) H3K27me3 signal in the parental MAC after fragmentation. (**C**) H3K27me3 signal in the developing MACs. Green: H3K27me3; blue: DAPI; m: parental macronucleus; dotted circles: developing macronucleus; scale bar: 10 μm. Control: cells silenced with empty L4440 vector, *NOWA1* KD: *NOWA1* knockdown, *PTCAF-1* KD: *PTCAF-1* knockdown.

As in *T. thermophila*, where H3K9me3 and H3K27me3 are required for heterochromatin formation prior to programmed DNA elimination (38,42) we observed the appearance of both these histone modifications during development in *P. tetraurelia*. Immunofluorescent labelling of control cells with H3K9me3- and H3K27me3-specific antibodies showed very low levels of these modifications in the vegetative MAC (Figures 5A and 6A). Higher levels of both modifications were detected in the fragments of the parental MAC at the beginning of sexual development (Figures 5B and 6B) and even higher levels were observed in the developing MACs (Figures 5C and 6C). Since PtCAF-1 has a histone binding domain, we were interested to see if it might be involved in the setting of these histone modifications. At the same time we were also interested to see if *NOWA1* KD would affect H3K9me3 and H3K27me3. No significant difference in H3K9me3/K27me3 level was detected in the parental MAC between control, *PTCAF-1* KD and *NOWA1* KD (Figures 5A and 6A). In the fragments of the old MAC the signals for H3K9me3/K27me3 were strongly reduced in the *PTCAF-1* KD and *NOWA1* KD cells (Figures 5B and 6B). In the developing macronuclei, where both modifications are normally present at the highest level, the signals in both knockdown experiments were also strongly reduced (Figures 5C and 6C). Although both knockdowns did not lead to 100% lethality in progeny cells, the changes of the levels of H3K9me3 and H3K27me3 were visible in all cells at the corresponding life stages. These observations suggest that the absence of either protein may lead to the reduction of H3K9me3/K27me3 in both parental fragments and the developing MACs.

**Figure 7.**
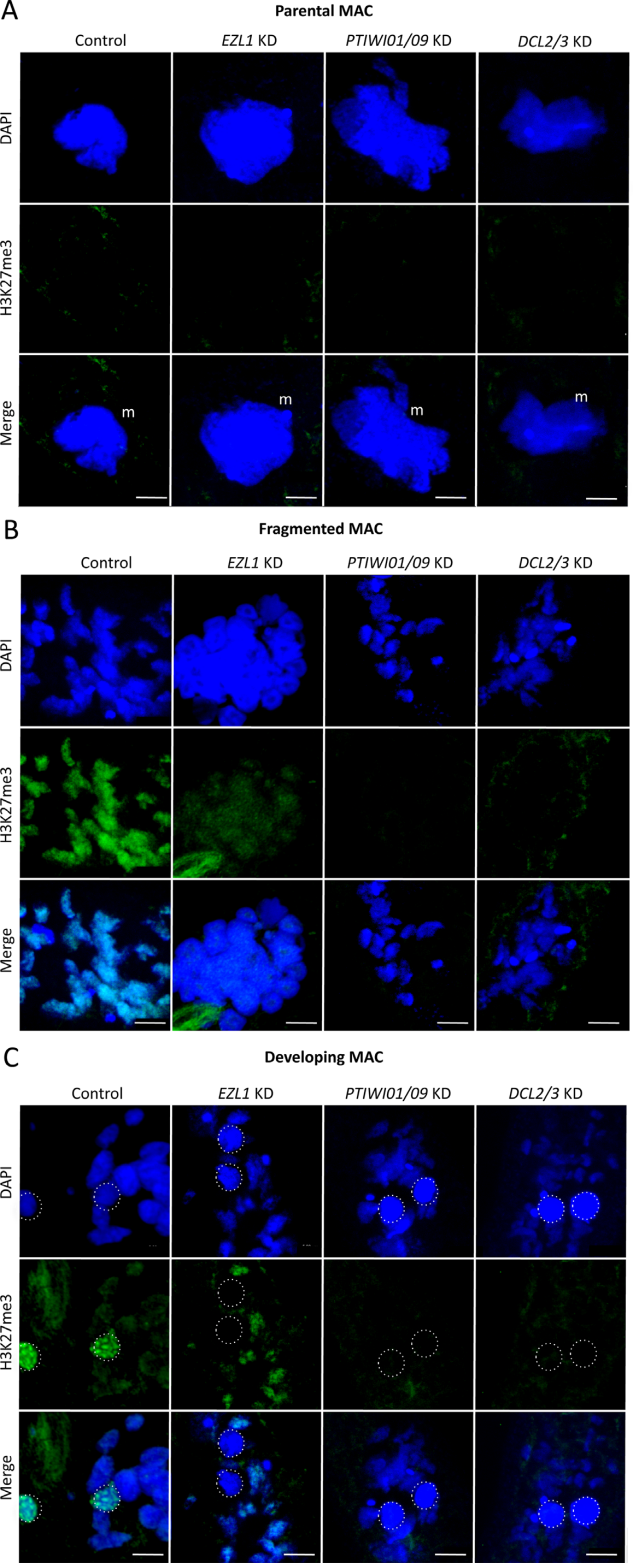
H3K27me3 in control, *EZL1* KD, *PTIWI01/09* KD and *DCL2/3* KD cells. (**A**) H3K27me3 signal in the parental MAC. (**B**) H3K27me3 signal in the parental MAC after fragmentation. (**C**) H3K27me3 signal in the developing MACs. Green: H3K27me3; blue: DAPI; m: parental MAC; dotted circles: developing MAC; scale bar: 10 μm. Control: cells fed with *Escherichia coli* expressing empty L4440 vector.

To verify the specificity of the H3K27me3 antibody we used *EZL1* KD cells. Ezl1 is the homologue of *Drosophila* E(z) and was described in *Tetrahymena* as developmentally-specific and responsible for H3K27me3 necessary for DNA elimination (38). After *EZL1* silencing in *Paramecium*, H3K27me3 is strongly reduced in the fragments of the parental MAC and is absent from developing MACs (Figure 7). Furthermore, investigation of both *PTIWI01/09* and *DCL2/3* double knockdown cells revealed the absence of H3K27me3 from the fragments of the parental MAC and from developing MACs (Figure 7).

Trimethylation of lysine 4 on histone 3 (H3K4me3), which is generally considered to be specific for active chromatin (50,51), is present in the intact parental MAC and the fragmented MAC in the control cells. Neither *PTCAF-1* nor *NOWA1* KD affects H3K4me3 methylation (Supplementary Figure S4). *DCL2/3* KD not only affects H3K27me3 (Figure 7) but also eliminates H3K4me3 signal during development (Supplementary Figure S4). In *PTIWI01/09* KD H3K4me3 is present in the parental MAC but is strongly decreased in the fragmented MAC (Supplementary Figure S4). To rule out the possibility that the amount of histone H3 was affected during knockdown we checked the levels of histone H3 by western blot during development (Supplementary Figure S5 A). The levels of histone H3 do not change in either the control or in the silenced samples showing that our results for H3K9me3 and H3K27me3 are not due to the absence of histone H3. The decrease of both these H3 trimethylation marks in *PTCAF-1* KD cells during development observed by immunostaining was verified by western blot (Supplementary Figure S5 B).

In conclusion, our results suggest that, like in *Tetrahymena*, these two histone modifications correlate with programmed DNA elimination in *Paramecium* and that the presence of both PtCAF-1 and Nowa1 is required for the assembly of these modifications during development.

## DISCUSSION

The development of a functional somatic nucleus in *Paramecium* includes genome-wide programmed DNA rearrangements. Excision of thousands of IESs and a number of transposable elements depends on a DNA targeting mechanism employing scnRNAs. In this study we identified a new protein that is necessary for the excision of the scnRNA-sensitive DNA (including maternally-controlled IESs and transposons). Though PtCAF-1 is expressed at the beginning of sexual development (Figure 1B and C), the PtCAF-1 GFP fusion protein persists in the cell beyond the beginning of development throughout all the subsequent developmental stages we examined (Figure 3). This observation and the impact of *PTCAF-1* knockdown on scnRNA population suggest that PtCAF-1 may have a dual role during development—one in the parental MAC and one in the developing MAC. At the beginning, when PtCAF-1 is present in the parental MAC, it may have a role in the selection of germline-specific scnRNAs. Later, when the protein localizes to the developing MAC it may play a role in scnRNA-mediated targeting of IES excision. The knockdown leads only to retention of mcIESs and transposons (which are also maternally controlled) and not to the retention of non-mcIESs. The effect of *PTCAF-1* knockdown on new MAC development is very similar to the effect of *NOWA1* knockdown, suggesting that these two proteins may be involved in the same pathway. We did not observe any morphological abnormalities during sexual development in *PTCAF-1* silenced cells, therefore lethality of sexual progeny cells could be attributed solely to the incomplete elimination of MIC genome-specific sequences from the developing MAC because most IESs interrupt sequences of protein coding genes. scnRNAs produced from the entire MIC genome match both mcIES and non-mcIES. Since depletion of the proteins responsible for scnRNA production (Dcl2/3) only strongly affects mcIESs (52), and likewised depletion of PtCAF-1 only appears to affect mcIESs, we suggest that PtCAF-1 depletion results in mcIES retention because it affects the scnRNA population. It is conceivable that non-mcIESs have distinct features within their sequences, allowing them to be excised by the PiggyMac endonuclease without prior targeting by scnRNAs, whereas mcIESs do require scnRNAs for their targeting and excision.

Our results indicate that the protein has a function in the epigenetic pathway leading to the maternally controlled excision of DNA. Chromatin assembly factor 1 subunit C (CAF-1C), also known as histone-binding protein RBBP4 or RBAP48, is found in several complexes involved in histone methylation (PRC2/EED-EZH2 complex) and histone deacetylation (HDAC complex), where it is likely to function as a scaffold promoting both protein–protein interactions within the complexes and complex–histone interactions (53). Since PtCAF-1 has both a histone binding domain and a WD-domain within a single polypeptide and is upregulated during development, it has the potential to direct histone modifying enzymes to histone H3, which would explain the strong reduction of H3K9me3/K27me3 at specific time points during development in PtCAF-1 depleted cells (Figures 5 and 6).

As in *T. thermophila* where histone modifications are set to establish heterochromatin prior to DNA elimination (38,42), our results suggest that histone modifications play a role in targeting DNA sequences for elimination in *P. tetraurelia*. We showed that PtCAF-1 was first present in the parental MAC and remained there during fragmentation (Figure 3). Simultaneously H3K9me3 and H3K27me3 appeared in the parental MAC (Figures 5B and 6B). The fragments of the parental MAC remain transcriptionally active (54) but we cannot rule out that histone modifications are used to inactivate certain genes whose expression is not required or need to be downregulated during development. However, we observed no change in the gene expression of the developmental-specific genes we tested (*PTIWI01/09*, *DCL2/3*) in *PTCAF-1* KD cells.

After silencing of *PTCAF-1* the levels of H3K9me3 and H3K27me3 were strongly reduced (Figures 5B and 6B). As no replication takes place in the parental MAC at this stage of development we can exclude that PtCAF-1 is involved in *de* *novo* nucleosome assembly as initially characterized for CAF-1 (55). One possible function of PtCAF-1 in the parental MAC could be the attraction recruitment of histone modifying enzymes that in turn would methylate histone H3 at lysine 9 and 27. Our results suggest that silencing of genes involved in the scnRNA pathway like *DCL2/3*, *PTIWI01/09* and *NOWA1/2*, lead to the absence of H3K27me3 and H3K9me3. This suggests that the deposition of these histone marks is due to scnRNAs binding to their targets in the parental MAC. As PtCAF-1 is required for the scanning and it carries a histone-binding domain, we believe that the scnRNA-related machinery interacts with chromatin during the scanning process.

We also showed that later during development PtCAF-1 concentrates in the developing MAC (Figure 3) which coincides with accumulation of H3K9me3 and H3K27me3 (Figures 5C and 6C). In *PTCAF-1* KD cells these methylation marks are strongly reduced in the developing MAC. Since at this stage a massive DNA endoreplication occurs, amplifying the genome up to 800 copies, PtCAF-1 could potentially be involved in *de* *novo* nucleosome assembly. However, if the presence of nucleosomes in the newly developing genome is essential for correct DNA elimination, it would be difficult to explain how only the subset of IESs normally requiring scnRNAs for their excision is retained after *PTCAF-1* knockdown. Clearly, the fact that only mcIESs are sensitive to the knockdown, suggests that it specifically disturbs the RNA-guided DNA elimination. It is possible that PtCAF-1 attracts histone modifications to label mcIESs for subsequent elimination by PiggyMac. We showed that the amount of histone H3 does not vary during development and is not affected in *PTCAF-1* KD or *NOWA1* KD cells (Supplementary Figure S5 A). Therefore we can exclude variation of H3 levels to be the reason for reduced H3K9me3 and H3K27me3 signals in *PTCAF-1* KD and *NOWA1* KD cultures. We have also shown that H3K4me3 localization patterns do not change in *PTCAF-1* KD and *NOWA1* KD cells compared to control cells. The difference between the effects of *DCL2/3* and *PTIWI01/09* KD versus *PTCAF-1* and *NOWA1* KD on H3K4me3 in the parental MAC (Supplementary Figure S4) may be due to the fact that in *DCL2/3* and *PTIWI01/09* silencing there's no scnRNAs present in the cells at all (20,21). Therefore it is possible that presence of scnRNAs and not their selection is required for this modification in the parental MAC. In case of *PTCAF-1* KD we showed that the expression of non-coding RNAs from the parental MAC is not affected (Supplementary Figure S3). As both scnRNAs and non-coding RNAs are present in *PTCAF-1* KD cells we conclude that the PtCAF-1 is not involved in the production of either but has a more direct function in the scanning process.

In *Tetrahymena* where IES sizes range around 1–2 kb (56,57) and their elimination is accomplished in an imprecise manner (58), the use of histone modification as marks for elimination seems quite plausible. In contrast, *Paramecium* IESs have a mean length of 28 bp and are eliminated precisely. In general, shorter *Paramecium* IESs have a lesser requirement for scnRNA targeting than longer ones and transposable elements (28). One can imagine a scenario where histone modifications highlight IES-containing genomic regions in an approximate fashion, which then attracts the excision machinery that can recognize IES ends and excise them precisely. Consequently, in future it will be important to determine how histone modifications might be used as marks to attract the excision machinery for the precise elimination of sequences much shorter than a nucleosome.

## SUPPLEMENTARY DATA

Supplementary Data are available at NAR Online.

SUPPLEMENTARY DATA

## ACCESSION NUMBERS

PDB IDs: XM_001457471, XM_001427355 and XM_001461420, PRJEB4978, ERR372239–ERR372250, ERR372001, SRR907874 and SRR907875.
