# Supplementary material for: Paramecium tetraurelia chromatin assembly factor-1-like protein PtCAF-1 is involved in RNA-mediated control of DNA elimination
Source: Nucleic Acids Res. 2014 Sep 30;42(19):11952–64. doi: 10.1093/nar/gku874 (PMC4231744; doi:10.1093/nar/gku874)
Supplement: SUPPLEMENTARY DATA [file supp_42_19_11952__index.html]

Paramecium tetraurelia chromatin assembly factor-1-like protein PtCAF-1 is involved in RNA-mediated control of DNA elimination — Paramecium tetraurelia chromatin assembly factor-1-like protein PtCAF-1 is involved in RNA-mediated control of DNA elimination — SUPPLEMENTARY DATA 

# *Paramecium tetraurelia* chromatin assembly factor-1-like protein PtCAF-1 is involved in RNA-mediated control of DNA elimination

## SUPPLEMENTARY DATA

**Files in this Data Supplement:**

- SUPPLEMENTARY DATA
