# Supplementary material for: Paramecium tetraurelia chromatin assembly factor-1-like protein PtCAF-1 is involved in RNA-mediated control of DNA elimination
Source: Nucleic Acids Res. 2014 Sep 30;42(19):11952–64. doi: 10.1093/nar/gku874 (PMC4231744; doi:10.1093/nar/gku874)
Supplement: SUPPLEMENTARY DATA [file supp_gku874_nar-02111-d-2014-File009.pdf]

Supplemental Data:

A

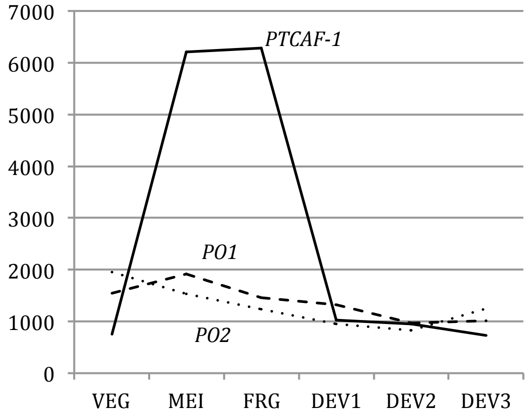

B

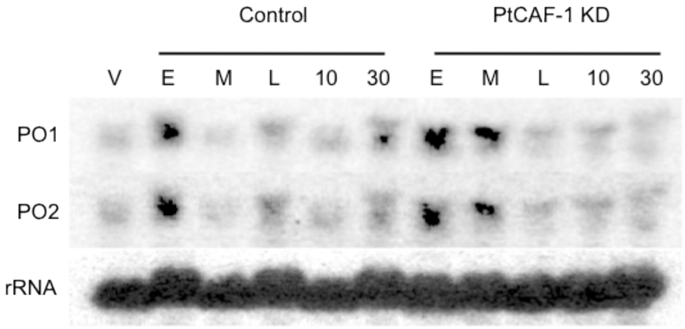

Figure S1. A Expression pattern of *PTCAF-1* ohnologs, *PO1* and *PO2* during autogamy (for details see Figure 1B). **B** Expression of *PO1* and *PO2* analysed by Northern blot. Cells during vegetative growth (V) and during autogamy where sampled from control and *PTCAF-1* KD cultures and analysed for *PO1* and *PO2* expression. E indicates a culture with 50% of cells with fragmented MAC (E ≈ FRG in B). M indicates a culture with 100% of cells with fragmented MAC (M ≈ between FRG and DEV1 in B). L indicates a culture 6 hours after M (L ≈ DEV1 in B). 10 and 30 indicate cultures 10 and 30 hours after M, respectively (10 ≈ DEV2 in B, 30 is ~ 10 h later than DEV3 in B). Consecutive hybridisations on the same membrane were performed with *PO1, PO2* and 17S rRNA probes.

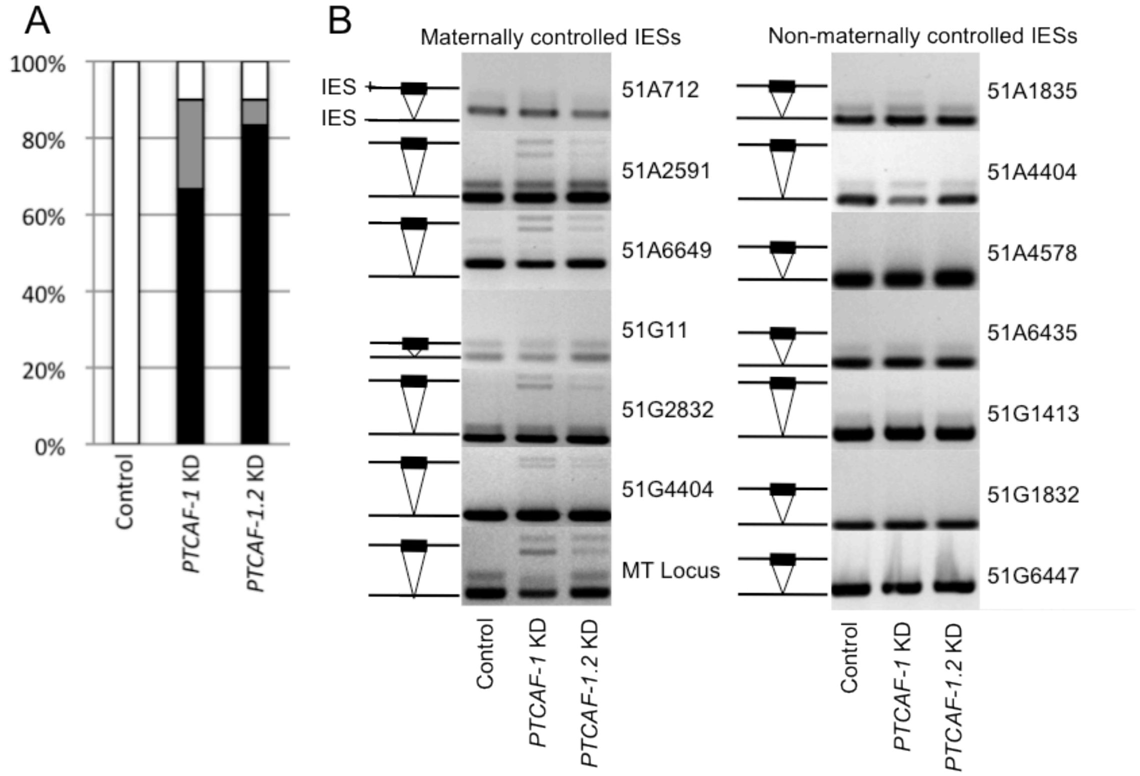

Figure S2. A Effect of *PTCAF-1* KD using an alternative silencing construct (*PTCAF-1.2*) on cell survival. After going through autogamy in silencing medium the cells where recovered in WGP medium. The graph shows the survival after three days. black: percentage of dead cells; grey: percentage of sick cells showing an altered division rate and behavior; white: percentage of cell growing at a normal rate. Silencing vector without insert was used as negative control. **B** Effect of *PtCAF-1* KD using an alternative silencing construct (*PTCAF-1.2*) on IES excision. IES retention was tested by PCR using primers flanking specific IESs sequences within the *51G* and *51A* genes as well as the mating-type locus (MT Locus). The excised form (IES-) is always detectable due to its presence in the fragments of the parental MAC. The unexcised form (IES+) is only detectable in case of retention in the newly developing macronuclei. Maternally controlled IESs: 51A712, 51A2591, 51A6649, 51G11, 51G2832, 51G4404, MT-Locus; non-maternally controlled IESs: 51A1835, 51A4404, 51A4578, 51A6435, 51G1413, 51G1832, 51G6447.

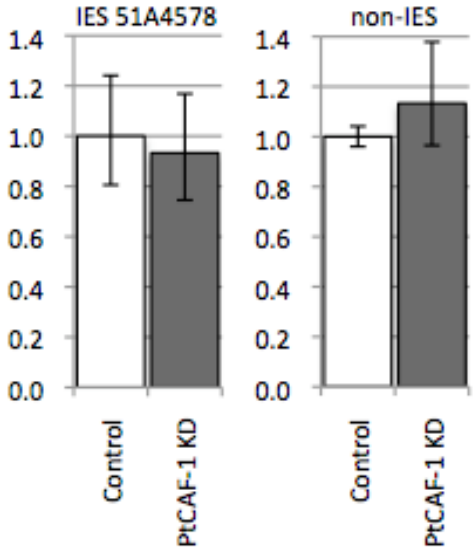

Figure S3. Expression of long non-coding RNAs containing an IES locus (IES 51A4578) and an intergenic locus (non-IES) at an early time point during development in control and *PTCAF-1* KD cells analysed by RT-qPCR. The data was normalized to the expression of *Paramecium* small and large rRNAs subunit genes. The respective control was used as calibrator and set as 1.

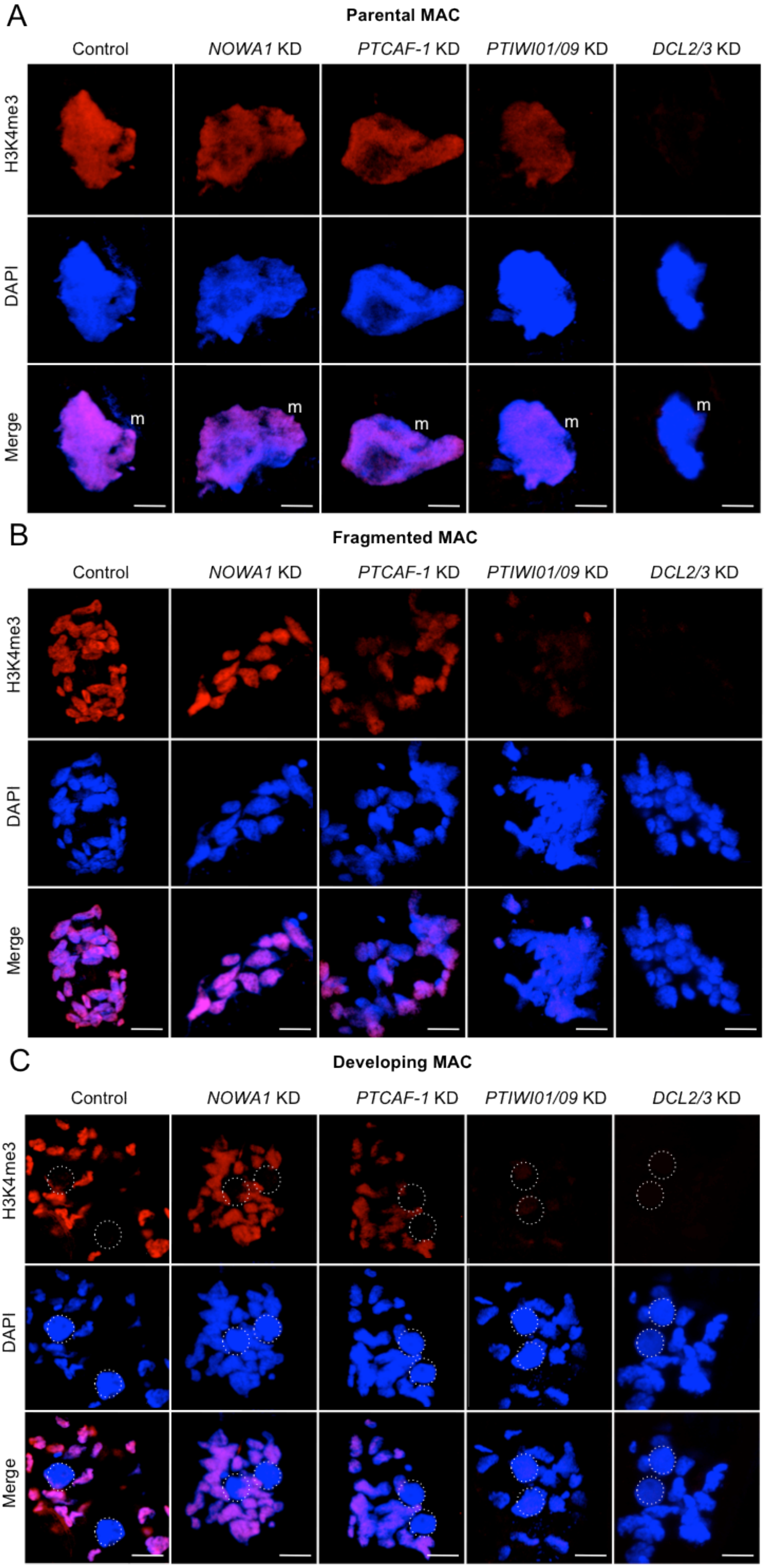
A
Parental MAC
Control
NOWA1 KD
PTCAF-1 KD
PTIWI01/09 KD
DCL2/3 KD
H3K4me3
DAPI
Merge
m
B
Fragmented MAC
C
Developing MAC

Figure S4. H3K4me3 in control, *NOWA1* KD, *PTCAF-1* KD, *PTIWI01/09* KD and *DCL2/3* KD cells. **A** H3K4me3 signal in the parental MAC. **B** H3K4me3 signal in the parental MAC after fragmentation. **C** H3K4me3 signal in the developing MACs. Red: H3K4me3; blue: DAPI; m: parental MAC; dotted circles: developing MAC; scale bar: 10 µm. Control: cells silenced with empty L4440 vector.

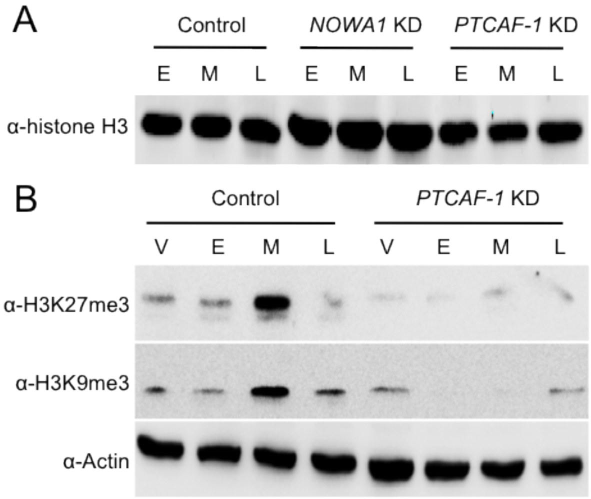

Figure S5. A Western blot with general anti histone H3 antibody on whole cell lysate of control, *NOWA1* KD and *PTCAF-1* KD cultures at specific time-points during development. Samples were prepared from the same number of cells. Transfer quality was assessed by Ponceau S staining (not shown). E: early (50% of cells with fragmented MAC); M: middle (100% of cells with fragmented MAC); L: late (6h after M). **B** Western blots analysis of H3K9me3 and H3K27me3 in control and *PTCAF-1* KD cells at specific time point during development. V: vegetative cells; E: early (50% of cells with fragmented MAC); M: middle (100% of cells with fragmented MAC); L: late (6h after M). Anti-actin antibody was used as loading control.

IES	Primer sequence (5' to 3' orientation)
51G-11F	ATCATAAGATTGATATCTTCTCCCTTCTCC
51G-11R	ACTTGCTACTAAAGCAAGAAACATTGAGAG
51G1413F	GAAGCTGCTTGTGTTAAGAATTCTACTGG
51G1413R	GCATCCAGCACTAGTTGAATTTACTGTAC
51G1832F	CTATAACTCTTGAAGCTGCTTGTAATATG
51G1832R	TTGTCAATGAGCCATTAACAGTTGCTGGAT
51G2832F	GAGCAGGATGTACAAATACTGGTGG
51G2832R	AGCTGATTAGATAACAATACAACCAGTACC
51G4404F	CTGTTGCTACACATTGTGCATATGTTACT
51G4404R	GCTGTAAGATTAACATTGAGCATGATCAAG
51G6447F	AATGCATCAAATGTAGTAACTACTCCTGCT
51G6447R	AATTTGTAAAGTATCCAGCGCAGGCAG
MT Locus F	GGTGTTTATATCTTAATTGTTGACCCTCAC
MT Locus R	CCATCTATACTCCATTCTTTATCTTAATTCAT
51A-712F	TTTGTCAAAAAGACATGTATCAAAATGCAG
51A-712R	TAGAATACTAAGAGATTCAATACAACAAAC
51A1835F	TAATGTATTGATAAGGCTTGCTCTACAGCC
51A1835R	ATCCTAACATCCTTGAATAGTTACTGATCC
51A2591F	ATGTGTTTGGACTGGATTGGCATGTAGAAG
51A2591R	GATGTAGCATAACATTTATCAACAATCCAT
51A4404F	TGGAATAGTGCTGCATCACCAGCTGCTTGC
51A4404R	CCAGTTATTGAACTGCAACTTACTGCAGTG
51A4578F	CACTGCAGTAAGTTGCAGTTCAATAACTGG
51A4578R	TGTAGTCTTAAAATCTTAGCATGTTGTACC
51A6435F	CAAATTGTGTCACTAGAGGTACATGTTTCC
51A6435R	GCGACATCAATAGTAACAGCTGAGCATGAG
51A6649F	ACTGCACCTCTAACTTTAACAAGCGAAGCA
51A6649R	CAGCAGTACATCCAGCTCTCTAAGTTTAGC

Table S1. Primers used in IES retention PCRs. F: forward primer; R: reverse primer

	total	25 nt	25 nt *51G*	25 nt *51G* r/m
Control E	33717933	12000476	745	22.09506733
PTCAF-1 E	32670471	13843135	805	24.63998759
Control L	17043918	1790359	49	2.874925824
PTCAF-1 L	18476116	7561296	339	18.3480121

Table S2. Library sizes of high-throughput sequencing of *P. tetraurelia* small RNAs for both time-points of control and *PTCAF-1* KD. Total library reads (total); 25 nt reads (25 nt); 25 nt reads mapping only to *51G* locus (25 nt *51G*); 25 nt reads per million, mapping only to *51G* locus, normalized to library size 25 nt *51G* r/m.

Supplemental Experimental Procedures

RT-qPCR of non-coding RNAs

RT-qPCR was performed by IMGM laboratories GmbH (Martinsried, Germany). *Paramecium* total RNA extracts from early and late time points during development of control and *PTCAF-1* KD cells were used for reverse transcription reactions with High-Capacity cDNA Reverse Transcription Kit (Applied Biosystems). qPCR Primer and TaqMan® MGB probes labeled with 6-FAM were designed for anti-sense transcripts of the IES 51A4578 locus and to the sense transcripts of an intergenic non-IES locus (MAC1>scaffold51_1:161800..163299). Primers and probes were also designed for *Paramecium* small (GenBank: X03772) and large (GenBank: EU828456) subunit rRNAs these were used as endogenous controls for data normalization. qPCR analysis was carried out in triplicates. Relative expression levels were calculated for the individual samples using ViiA7 Software v1.2.2 (Applied Biosystems).

Western Blot

Total protein was extracted according to the protocol for TRI® Reagent (T9424, Sigma) and quantified with Pierce™ BCA Protein Assay Kit (23225, Thermo Scientific). The total protein samples were separated electrophoretically on a 12% SDS-PAGE gel. Proteins were transferred onto a 0.2 µm nitrocellulose membrane (Whatman, Protran™ BA83). The membrane was blocked overnight at 4°C with PBS 5% BSA. Membranes were incubated overnight with primary antibodies (anti-H3K9me3, 1:5000, Millipore, cat: 07-442, lot: 2120113 or anti-H3K27me3, 1:5000, Millipore, cat: 07-449, lot: 2275589 or anti-histone H3, 1:5000, Abcam, cat: ab1791, lot: GR103809 or anti-Actin, 1:5000, Sigma, cat: A5060) in PBS 5% BSA + 0.2% Tween-20 at 4°C and then washed four times for 15 min with PBS 0.2% Tween-20. The membrane was then incubated for 1 h at RT with the secondary antibody (1:5000, goat anti-rabbit HRP conjugate, SantaCruz, sc-2004) diluted in PBS 5% BSA + 0.1% Tween-20. The membrane was then washed four times for 15 min with PBS 0.2% Tween-20 and once with PBS for 5 minutes. 500 µl of Luminata HRP substrate (WBLURO100, Millipore) was applied onto the membrane. The membrane was then scanned with Chemiluminiscent system (Fujifilm). The blots were analyzed using AIDA software.
